# Partial genetic suppression of a loss-of-function mutant of the neuronal ceroid lipofuscinosis-associated protease TPP1 in *Dictyostelium discoideum*

**DOI:** 10.1242/dmm.018820

**Published:** 2014-12-24

**Authors:** Jonathan E. Phillips, Richard H. Gomer

**Affiliations:** Department of Biology, Texas A&M University, College Station, TX 77843-3474, USA.

**Keywords:** Neuronal ceroid lipofuscinosis, Batten disease, TPP1, Tripeptidyl peptidase 1, *Dictyostelium*

## Abstract

Neuronal ceroid lipofuscinosis (NCL) is the most common childhood-onset neurodegenerative disease. NCL is inevitably fatal, and there is currently no treatment available. Children with NCL show a progressive decline in movement, vision and mental abilities, and an accumulation of autofluorescent deposits in neurons and other cell types. Late-infantile NCL is caused by mutations in the lysosomal protease tripeptidyl peptidase 1 (*TPP1*). TPP1 cleaves tripeptides from the N-terminus of proteins *in vitro,* but little is known about the physiological function of TPP1. TPP1 shows wide conservation in vertebrates but it is not found in *Drosophila*, *Caenorhabditis elegans* or *Saccharomyces cerevisiae*. Here, we characterize ddTpp1, a TPP1 ortholog present in the social amoeba *Dictyostelium discoideum*. Lysates from cells lacking ddTpp1 show a reduced but not abolished ability to cleave a TPP1 substrate, suggesting that other *Dictyostelium* enzymes can perform this cleavage. ddTpp1 and human TPP1 localize to the lysosome in *Dictyostelium*, indicating conserved function and trafficking. Cells that lack ddTpp1 show precocious multicellular development and a reduced ability to form spores during development. When cultured in autophagy-stimulating conditions, cells lacking ddTpp1 rapidly decrease in size and are less viable than wild-type cells, suggesting that one function of ddTpp1 could be to limit autophagy. Cells that lack ddTpp1 exhibit strongly impaired development in the presence of the lysosome-perturbing drug chloroquine, and this phenotype can be suppressed through a secondary mutation in the gene that we name suppressor of *tpp1*^−^ A (*stpA*), which encodes a protein with some similarity to mammalian oxysterol-binding proteins (OSBPs). Taken together, these results suggest that targeting specific proteins could be a viable way to suppress the effects of loss of TPP1 function.

## INTRODUCTION

Neuronal ceroid lipofuscinosis (NCL), or Batten disease, is the most common childhood-onset neurodegenerative disease, and it is characterized by a progressive decline in vision, motor skills and mental ability (Jalanko and Braulke, 2009). The disease is inevitably fatal, with death generally occurring within a few years of the onset of symptoms, and there is currently no treatment available. The occurrence of NCL is one to five per 100,000 births (Zeman, 1974), with the highest incidence rate among Northern European populations (Gardiner et al., 1990; Yan et al., 1993), and an estimated 25,000 families in the United States are affected by NCL (Zhong et al., 2000). Children with NCL have autofluorescent bodies within lysosomes in both neurons and in non-neural tissues (Miley et al., 1978); however, whether these bodies are causative for the disease or are merely correlative is unclear (Warrier et al., 2013). In many but not all types of NCL, the primary protein component of these bodies is mitochondrial ATPase subunit c, which can constitute up to two-thirds of the mass of the fluorescent bodies (Palmer et al., 1989). Most types of NCL show an autosomal recessive pattern of inheritance, and mutations in any of the 14 characterized genes can lead to the development of NCL (Warrier et al., 2013). Many of the proteins encoded by these genes localize to and/or function in the lysosome (Palmer et al., 2013). Despite this lysosomal association, little is known about how mutations in this set of genes cause the symptoms associated with NCL.

Late infantile neuronal ceroid lipofuscinosis (LINCL), a subclass of NCL with an onset age of 2 to 4 years (Schulz et al., 2013), is caused primarily by mutations in the lysosomal peptidase tripeptidyl peptidase 1, or TPP1 (Sleat et al., 1997). TPP1, also known as CLN2, cleaves tripeptides from the N-terminus of proteins (Vines and Warburton, 1998) and shows optimal peptidase activity at pH 3.5 (Sohar et al., 1999). Fibroblasts from individuals with LINCL show a dramatic reduction in lysosomal TPP1 activity, with 2 to 6% activity of that of fibroblasts from unaffected individuals (Vines and Warburton, 1999). Purified TPP1 cleaves peptides corresponding to the sequences of mitochondrial ATPase subunit c, amyloid β, angiotensin II and substance P (Junaid et al., 2000), although *in vivo* substrates have not been identified. Little is known about the normal physiological functions of TPP1. TPP1 is highly conserved among vertebrates (Wlodawer et al., 2003), although TPP1 orthologs have not been detected in *Drosophila*, *Caenorhabditis elegans* or *Saccharomyces cerevisiae*, greatly limiting the study of TPP1 in model organisms.

The social amoeba *Dictyostelium discoideum* has been utilized to characterize genes that play a role in neurodegenerative diseases, such as Huntington’s disease (Myre et al., 2011), Alzheimer’s disease (Kim et al., 2009) and Parkinson’s disease (van Egmond et al., 2008). Under conditions where nutrients are abundant, *Dictyostelium* exists as a unicellular amoeba that feeds on bacteria (Kessin, 2001). However, when starved, *Dictyostelium* cells stop proliferating and begin secreting relayed pulses of the chemoattractant cyclic (c)AMP, leading to the aggregation of cells into groups of approximately 10^5^ cells (Firtel and Meili, 2000). These groups then undergo a morphogenetic process that produces a fruiting body structure, which comprises an approximately 2-mm stalk supporting a ball of spores, ~24 hours after the onset of starvation (Loomis, 1982). Despite the considerable evolutionary distance between *Dictyostelium* and humans, the tractable genetics and biochemistry of *Dictyostelium* have proven useful for the study of genes that are associated with neurodegenerative diseases (Annesley et al., 2014). Recently, a report on the *Dictyostelium* ortholog of CLN3, another gene that can underlie NCL pathology when mutated, has revealed that *Dictyostelium* cells that lack CLN3 show precocious development (Huber et al., 2014), indicating that CLN3 functions as a negative regulator in *Dictyostelium*. Expression of human CLN3 in this mutant rescued this phenotype, demonstrating that CLN3 is functionally conserved and that the study of NCL-associated genes in *Dictyostelium* might help us to understand the physiological functions of these genes and how mutations in these genes are linked to NCL pathologies.

**TRANSLATIONAL IMPACT**

**Clinical issue**

Neuronal ceroid lipofuscinosis (NCL) is a childhood-onset neurodegenerative disease that is inevitably fatal and has no cure. The disease is caused by genetic mutations in any of 14 characterized genes, all of which result in a similar class of symptoms, including progressive decline in vision, motor functions and mental ability. A better understanding of the function of these genes might guide the development of therapies. One of these genes, *CLN2*, encodes the lysosomal protein tripeptidyl-peptidase 1 (TPP1). Little is known about the normal physiological function of TPP1, in part because TPP1 is not present in many commonly used model organisms, such as yeast, fruit flies or roundworms. However, TPP1 is present in *Dictyostelium discoideum*, a well-studied and genetically tractable amoeba.

**Results**

In this work, the authors characterize ddTpp1, the *Dictyostelium* ortholog of TPP1, and show that ddTpp1 has multiple similarities to the human protein in proteolytic activity and trafficking. *Dictyostelium* cells that are mutant for TPP1 (*ddTpp1* disruption mutant*, tpp1*^−^) are less viable than wild-type cells under conditions that induce autophagy, suggesting that TPP1 plays a role in the autophagic process. In addition, the authors find that a secondary mutation in the gene that they name suppressor of *tpp1*^−^ A (*stpA*), which encodes a protein with similarity to a human protein involved in sphingolipid metabolism, can suppress a phenotype that is associated with a lack of ddTpp1, suggesting that perturbing the function of analogous human suppressor genes might ameliorate some of the symptoms induced by mutation of TPP1.

**Implications and future directions**

This study establishes *Dictyostelium* as a tractable model system for the study of NCL caused by mutation of TPP1. Future directions include utilizing *Dictyostelium* to better understand the normal physiological roles of TPP1 and the use of *Dictyostelium* genetics to identify novel genetic suppressors of phenotypes caused by loss of TPP1 function. Identifying such suppressors could guide approaches to the treatment of NCL pathologies.

Here, we report that ddTpp1, the *Dictyostelium* ortholog of TPP1, shows multiple functional similarities with human TPP1. Cells that lack ddTpp1 (*tpp1*^−^) show a reduced but not abolished ability to cleave the synthetic TPP1 substrate Ala-Ala-Phe-amino-methylcoumarin (AAF-AMC). ddTpp1 and human TPP1 localize to the lysosome when expressed in *Dictyostelium*, indicating conserved lysosomal function and trafficking mechanisms. Previous work has shown that ddTpp1 expression is detectable only during development (Iranfar et al., 2001). Consistent with this expression pattern, we find that *tpp1^−^* cells resemble wild-type cells during vegetative growth but show precocious development and a reduced ability to generate spores during development. Further, starved *tpp1*^−^ cells show intracellular autofluorescent bodies that are analogous to those seen in the cells of individuals that lack TPP1. In response to amino acid starvation, *tpp1^−^* cells show reduced cell size and viability as compared with wild-type cells, suggesting that autophagy is aberrant in *tpp1*^−^ cells. The development of *tpp1*^−^ cells is strongly impaired in the presence of the lysosome-perturbing compound chloroquine, and this phenotype can be suppressed through a secondary mutation in a gene that we name suppressor of *tpp1*^−^ A (*stpA*), a gene encoding a protein with similarity to mammalian oxysterol-binding proteins (OSBPs).

## RESULTS

The *Dictyostelium* genome encodes a putative TPP1 ortholog (ddTpp1) with 37% identity and 52% similarity to the human TPP1 protein, with 100% conservation at catalytic residues (supplementary material Fig. S1) (Walus et al., 2010). Like human TPP1, *ddTpp1* encodes an N-terminal pro-peptidase activation domain and a C-terminal peptidase domain of the S53 family (Golabek and Kida, 2006; Van de Ven et al., 1993). *ddTpp1* is expressed at very low levels in vegetative cells, but is strongly upregulated during development in prespore cells (Iranfar et al., 2001), with expression peaking at 16 hours after the initiation of starvation (Parikh et al., 2010). To characterize ddTpp1, we sought to disrupt the *ddTpp1* gene. We used homologous recombination to generate a *ddTpp1* disruption mutant (*tpp1*^−^, see Materials and Methods) in the Ax2 wild-type background and confirmed disruption of the *ddTpp1* gene by using PCR analyses (supplementary material Fig. S2).

**Fig. 1.**
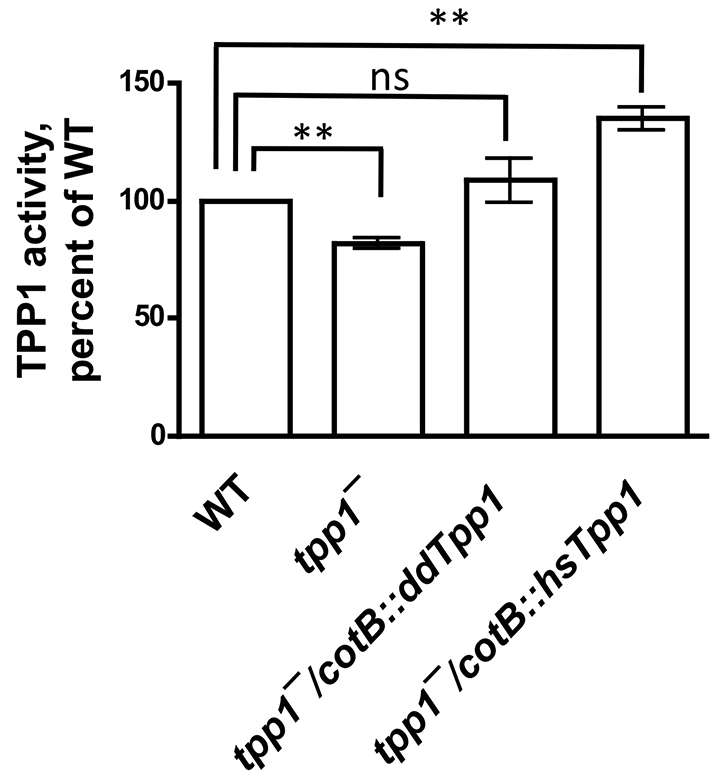
***tpp1^−^* cells show reduced proteolytic activity.** Cell lysates from equivalent numbers of 16-hour starved cells were incubated with 50 μM AAF-AMC, and the fluorescence of the released fluorophore AMC was measured 30 minutes after the addition of substrate. The fluorescence was normalized to that of wild type for each independent experiment. Values are means±SEM, *n*=4. **P<0.01; ns, not significant (one-sample *t*-test with comparison to the value 100%). hsTpp1, human TPP1.

The proteolytic activity of TPP1 can be measured using the substrate AAF-AMC, which changes fluorescence when the AAF tripeptide is removed through cleavage (Lin et al., 2001). To test whether the *tpp1*^−^ mutant shows reduced proteolytic activity, we assayed cell lysates from cells that had been starved for 16 hours for the ability to cleave this substrate. Lysates from *tpp1*^−^ cells showed somewhat reduced TPP1 activity as compared with wild-type cell lysates (Fig. 1). To attempt to rescue the reduced TPP1 activity in *tpp1*^−^ cells, we utilized the *cotB* promoter, which drives gene expression during development in prespore cells, resembling the endogenous ddTpp1 expression pattern (Zhang et al., 1999). Expression of ddTpp1 or human TPP1 in *tpp1*^−^ cells under control of the *cotB* promoter rescued the reduction in TPP1 activity that was seen in *tpp1^−^* cells (Fig. 1). These results indicate that ddTpp1 has proteolytic activity similar to that of human TPP1. We were unable to generate a strain in which the entire *ddTpp1* open reading frame was deleted, so we are unable to distinguish whether the TPP1 activity seen in the *tpp1*^−^ strain was due to other proteins that have enzymatic activity like that of TPP1, or whether the *tpp1*^−^ strain contains a hypomorphic allele with some retention of proteolytic activity. Regardless, these results indicate that the *tpp1*^−^ strain shows, at least, a partial loss of ddTpp1 activity.

**Fig. 2.**
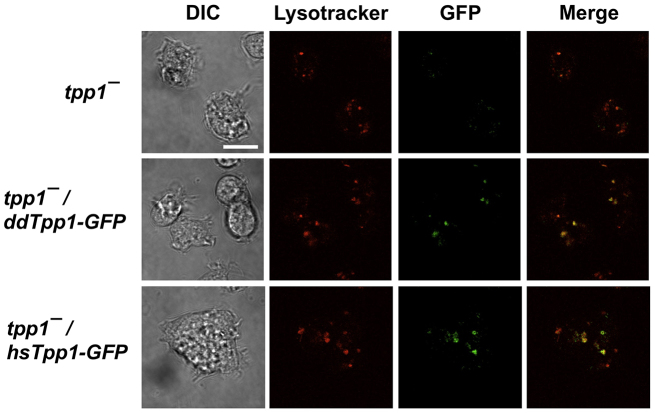
***Dictyostelium* and human TPP1 localize to the lysosome in *Dictyostelium* cells.** Cells were starved for 16 hours in shaking culture, stained with Lysotracker, and then imaged in chamber slides by confocal microscopy; images are representative of three independent experiments. hsTpp1-GFP, human TPP1 fused to GFP. Scale bar: 10 μm.

TPP1 is a lysosomal protease (Golabek and Kida, 2006). To examine whether ddTpp1 might also be a lysosomal protein, we constructed a protein comprising ddTpp1 fused to green fluorescent protein (ddTpp1-GFP) and examined the subcellular localization of this fusion protein. ddTpp1-GFP colocalized with the lysosomal marker Lysotracker (Fig. 2) and was not observed outside of lysosomes, indicating that ddTpp1 is associated with lysosomes. Some lysosomes, however, did not appear to contain ddTpp1-GFP. Human TPP1 also localized to the lysosome in *Dictyostelium* (Fig. 2), indicating that the trafficking mechanisms of these two proteins are conserved.

**Fig. 3.**
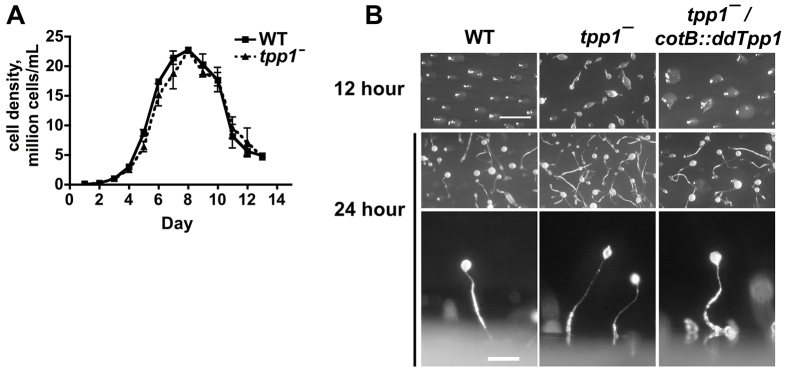
***ddTpp1* affects developmental timing but does not affect proliferation during vegetative growth.** (A) Cells were inoculated into HL5 medium at 1×10^5^ cells/ml, and cell density was determined daily with a hemocytometer. Values are mean±SEM, *n*=3. (B) Cells were spread onto non-nutrient agar plates and allowed to develop for the indicated time; images are representative of three independent experiments. Scale bars: 1 mm (overhead view); 200 μm (fruiting body side view).

Having shown that the human and *Dictyostelium* TPP1 orthologs exhibit conservation in function and subcellular localization, we next focused specifically on understanding the physiological roles of ddTpp1 in *Dictyostelium*. *ddTpp1* is strongly upregulated during development (Parikh et al., 2010), suggesting that ddTpp1 functions primarily at this stage. During vegetative growth, *tpp1*^−^ cells proliferate like wild-type cells (Fig. 3A), further suggesting that ddTpp1 functions primarily during development. To assess whether ddTpp1 affects development, we examined the developmental timing and morphology of wild-type and *tpp1*^−^ cells on non-nutrient agar plates. At 12 hours, wild-type cells had aggregated to form mounds (Fig. 3B). By contrast, *tpp1*^−^ cells showed precocious development, with a mixture of mounds, tipped mounds and migratory slugs. Expression of *ddTpp1* using the *cotB* promoter (*cotB::ddTpp1*) in the *tpp1*^−^ mutant rescued this precocious development. At 24 hours, wild-type, *tpp1*^−^ and *tpp1*^−^*/cotB::ddTpp1* cells had differentiated into fruiting bodies that showed normal morphology (Fig. 3B). These results indicate that ddTpp1 might play a role in developmental timing, but that ddTpp1 is not required for proper fruiting body morphogenesis.

**Fig. 4.**
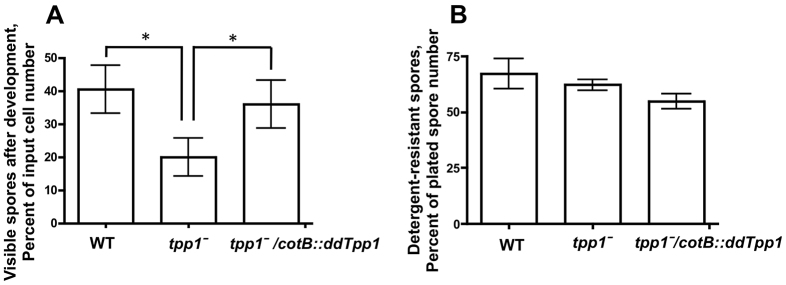
**Tpp1 affects the development of spores.** (A) Ten million cells were allowed to develop on filter pads for 24 hours, and then spores were collected and counted. (B) Equivalent numbers of the collected spores were treated with detergent and plated onto a lawn of *K. aerogenes* bacteria, and the number of resultant colonies was counted. Values are means±SEM, *n*=4. *P<0.05 (one-way ANOVA, Tukey’s test).

Because the temporal expression of *ddTpp1* suggests a role in the developmental process, we next tested whether ddTPP1 affects the number of viable spores that are generated during development by allowing a fixed number of cells to undergo development, collecting and counting the generated spores, and then plating spores onto lawns of bacteria and counting the resultant colonies from spore germination. *tpp1*^−^ cells produced significantly fewer visible spores than wild-type cells during development, and this phenotype could be rescued through expression of *cotB::ddTpp1* in *tpp1*^−^ cells (Fig. 4). We then plated equivalent numbers of detergent-treated spores onto lawns of bacteria and saw that there was no significant difference in the number of germinated spores when comparing wild-type, *tpp1*^−^ and *tpp1*^−^*/cotB::ddTpp1* cells. These results indicate that ddTpp1 affects the number of spores generated during development, but not spore viability.

**Fig. 5.**
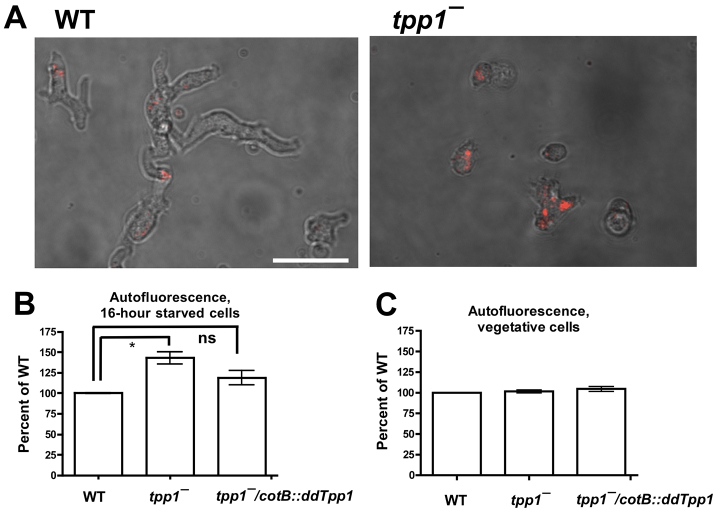
***tpp1*^−^ cells show increased intracellular autofluorescent material.** (A) Cells were starved for 16 hours in chamber slides, and intracellular autofluorescent material in cells at the periphery of cell aggregates was imaged by using confocal microscopy; images are representative of three independent experiments. Scale bar: 20 μm. (B) Cells were starved in shaking culture for 16 hours, and the cellular autofluorescence was assayed by using flow cytometry. The average fluorescence per cell was normalized to that of wild type. Values are means±SEM, *n*=3. *P<0.05; ns, indicates not significant (one-sample *t*-test with comparison to the value 100%). (C) Cells growing on a bacterial lawn were collected and assayed as described in B. Values are means±SEM, *n*=3. The differences between wild-type and either *tpp1*^−^ or *tpp1*^−^*/cotB::ddTpp1* are not significant (one-sample *t*-test with comparison to the value 100%).

Intracellular autofluorescent deposits are a hallmark of NCL (Golabek and Kida, 2006). To examine whether *tpp1*^−^ cells show similar accumulations of autofluorescent material, we examined the autofluorescence of cells by using fluorescence microscopy and flow cytometry. We allowed cells to undergo aggregation in chamber slides for 16 hours and then imaged cells at the periphery of aggregates. *tpp1*^−^ cells tended to show a more rounded morphology than wild-type cells and showed a higher degree of intracellular autofluorescence (Fig. 5A). Cells within the 16-hour aggregates showed significant levels of autofluorescence for both wild-type and ^−^ genotypes (data not shown). As a parallel approach, we starved cells for 16 hours in shaking culture and examined the autofluorescence of cells by using flow cytometry. *tpp1*^−^ cells were more autofluorescent than wild-type cells, and this phenotype could be rescued by expression of *cotB::ddTpp1* in the *tpp1*^−^ background (Fig. 5B). Wild-type and *tpp1*^−^ cells that had been collected from vegetative growth conditions on bacterial lawns showed similar levels of autofluorescence (Fig. 5C), indicating that the difference in autofluorescence was specific to the developmental phase. These results indicate that, as in humans, loss of TPP1 activity in *Dictyostelium* results in the accumulation of autofluorescent bodies within cells, although whether this autofluorescence derives from the same molecules in *Dictyostelium* as in individuals with NCL is currently unclear.

**Fig. 6.**
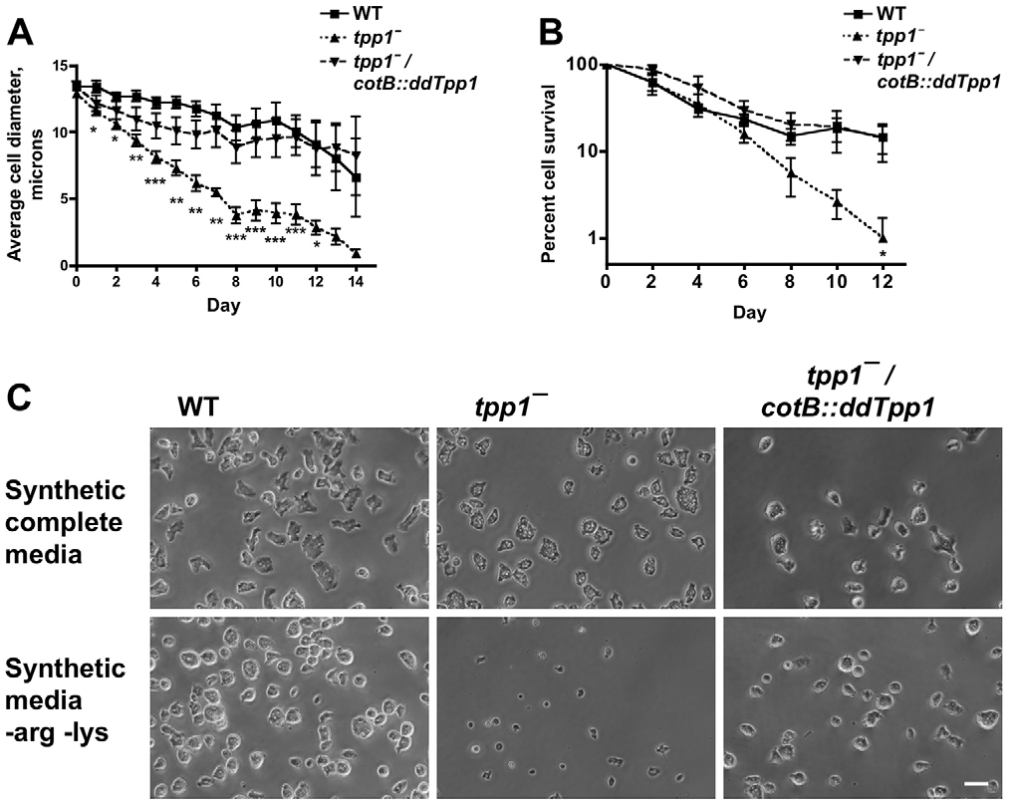
**Tpp1 affects the autophagic response of cells.** (A) The size of cells cultured continuously in autophagy-stimulating media (ASM) was measured daily by flow cytometry using forward scatter. Values are means±SEM, *n*=5. * indicates that differences between wild type and *tpp1*^−^ were significant with *P*<0.05, **P<0.01 and ***P<0.001 (one-way ANOVA, Tukey’s test). Differences between wild type and *tpp1*^−^*/cotB::ddTpp1* were not significant for any time point. (B) Every 2 days, an aliquot was taken from a culture of cells continually cultured in ASM and then spread onto a lawn of *K. aerogenes* bacteria, and the viability of cells was measured by counting the resulting colonies. Values are means±SEM, *n*=4. * indicates that differences between wild type and *tpp1*^−^ were significant with *P*<0.05 (one-way matched values ANOVA, Tukey’s test). Differences between wild type and *tpp1*^−^*/cotB::ddTpp1* were not significant for any time point. (C) Cells cultured for 8 days in ASM were allowed to settle in a glass chamber slide, and then cells were imaged with an inverted phase-contrast microscope; images are representative of three independent experiments. Scale bar: 20 μm.

The fact that *tpp1*^−^ cells show a deficiency in spore production during development indicates that the lysosomal protease ddTpp1 has some fitness-increasing function during development. Autophagy, the catabolism of sequestered cellular components via lysosomal fusion (Klionsky and Emr, 2000), is required for proper development in *Dictyostelium* (Otto et al., 2003). Autophagy can be stimulated in *Dictyostelium* by culturing cells in axenic autophagy-stimulating media (ASM) without the essential amino acids arginine and lysine, which results in a progressive reduction in cell size and protein content due to catabolism (King et al., 2011). In contrast to wild type, mutants defective in autophagy do not show a significant reduction in size in ASM (King et al., 2013). Further, autophagy-defective mutants show a marked reduction in protein turnover in autophagy-stimulating conditions as compared to wild type and are less viable than wild type when cultured in ASM, suggesting a loss in viability due to an inability to generate nutrients through catabolism (King et al., 2013). To test whether ddTpp1 affects the autophagic response of cells, we cultured cells in ASM and measured cell size and cell viability as a function of time. Wild-type and *tpp1*^−^ cells showed a similar initial size and decreased in size during concomitant arginine and lysine starvation, indicating that *tpp1*^−^ cells are capable of an autophagic response (Fig. 6A). However, *tpp1*^−^ cells were significantly smaller than wild-type cells when continuously cultured in ASM (Fig. 6A,C). Further, *tpp1*^−^ cells were less viable after culture in ASM, as assayed by the ability to form colonies on bacterial lawns (Fig. 6B). Both the cell size and cell viability phenotypes of *tpp1*^−^ cells in ASM were rescued by expression of *cotB::ddTpp1* in Tpp1 cells (Fig. 6), indicating that these phenotypes are due specifically to a deficiency of ddTpp1, and suggesting that developmentally regulated promoters are active under these conditions. Taken together, these results indicate that ddTpp1 is required for a proper autophagic response during amino acid starvation.

**Fig. 7.**
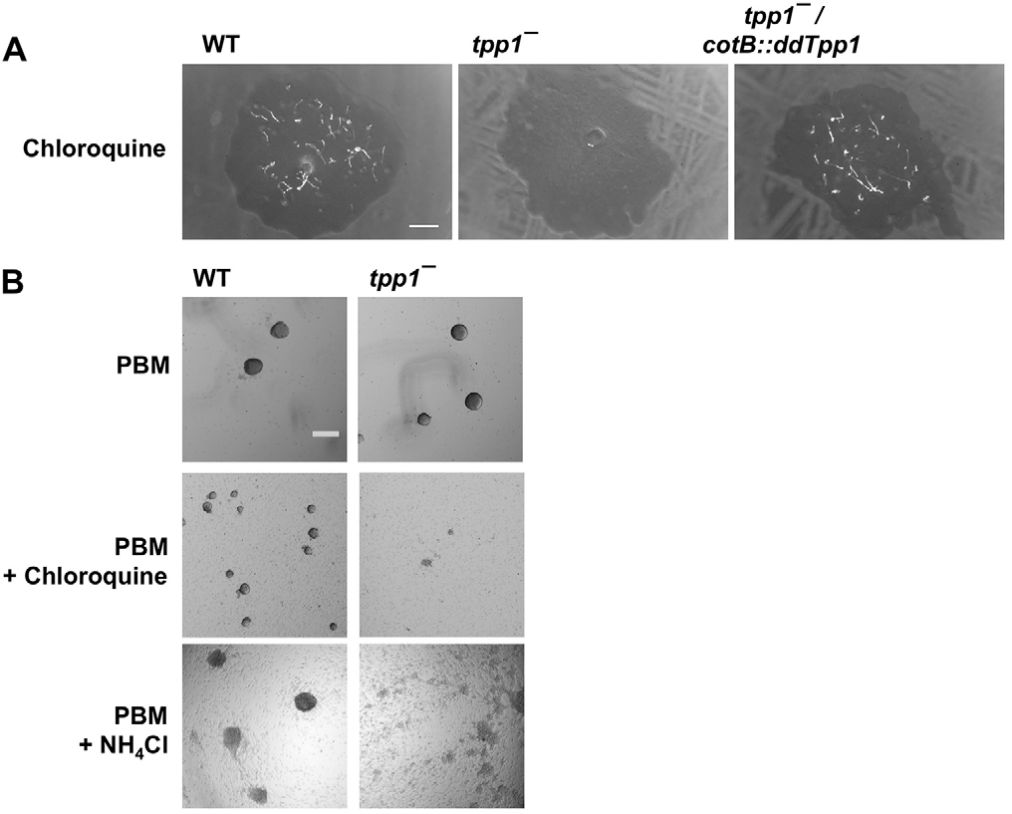
**Development of *tpp1*^−^ cells is inhibited in the presence of chloroquine or ammonium chloride.** (A) Clonal cell colonies were grown on SM/5 plates supplemented with 3 mM chloroquine and then spread with a lawn of *K. aerogenes* bacteria; images were taken after 8 days and are representative of three independent experiments. (B) Cells were starved in a 24-well plate for 14 hours in PBM (for PBM buffer components, see Materials and Methods), PBM with 5 mM chloroquine, or PBM with 20 mM ammonium chloride. All images are representative of three independent experiments. Scale bars: 1 mm (A); 200 μm (B).

Identifying second-site mutations that suppress the phenotypes of *tpp1*^−^ cells could guide approaches to reducing or preventing pathologies that arise from the loss of TPP1 activity in humans. We thus sought to identify phenotypes that we could utilize in a tractable screen for second-site suppressor mutations. As the *tpp1*^−^ mutant shows some tripeptidyl-peptidase activity, but less activity than wild type (Fig. 1), we reasoned that if some compound caused a developmental defect by affecting tripeptidyl-peptidase activity, the *tpp1*^−^ mutant should be more sensitive to such an effect than the wild type. While examining the effects of various compounds on wild type and *tpp1^−^* development, we saw that the drug chloroquine strongly impaired the development of *tpp1^−^* cells that were growing as colonies on a bacterial lawn (Fig. 7A). By contrast, the effects of chloroquine on the development of wild type were much less severe (Fig. 7A). Intriguingly, chloroquine, a weak base, is a well-characterized lysosomal inhibitor that accumulates in lysosomes due to protonation at low pH (Seglen et al., 1979) and that affects the regulation of autophagic flux (Li et al., 2013). We further tested the effect of chloroquine on development by examining the aggregation of cells in submerged culture and found that *tpp1*^−^ cells aggregated more slowly than wild-type cells in the presence of chloroquine (Fig. 7B). Ammonium chloride, another weak base that inhibits lysosomal function (Amenta and Brocher, 1980), also slowed aggregation and had a stronger effect on *tpp1*^−^ cells than on wild-type cells (Fig. 7B). These results indicate that the loss of ddTpp1 results in a hypersensitivity to the effects of chloroquine and ammonium chloride.

**Fig. 8.**
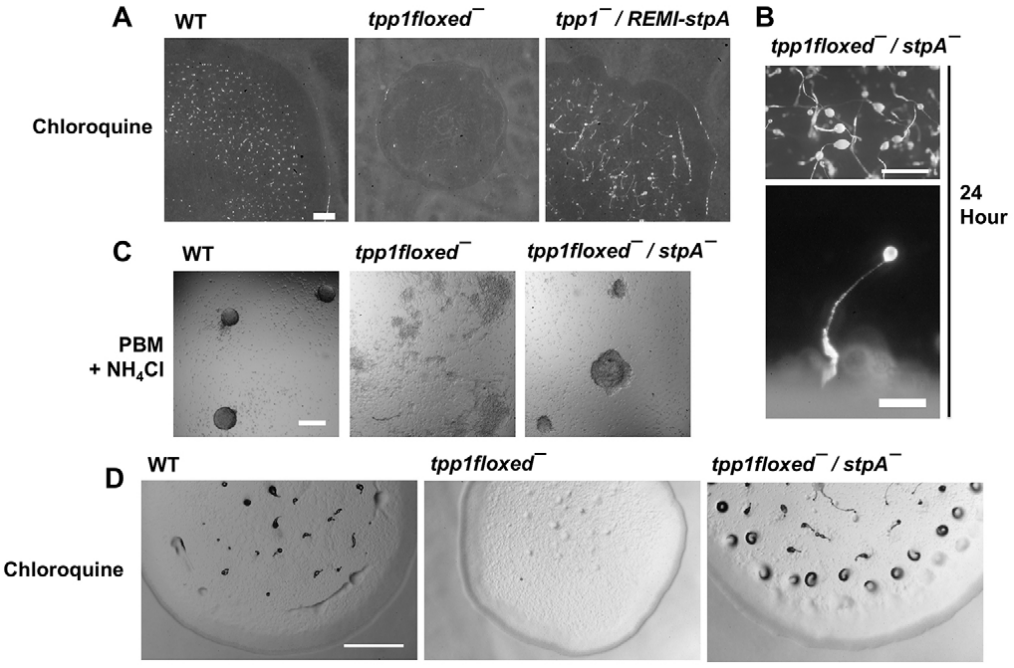
**Loss of StpA suppresses the slow development phenotype of *tpp1floxed*^−^ cells in the presence of chloroquine or ammonium chloride.** (A) Clonal cell colonies were grown on SM/5 plates supplemented with 3 mM chloroquine spread with a lawn of *K. aerogenes* bacteria; images were taken after 8 days. (B) *tpp1floxed^−^/stpA*^−^ development at 24 hours was examined as described in Fig. 3. (C) Cells were starved for 14 hours in a 384-well plate in PBM containing 20 mM ammonium chloride. (D) Development in the presence of chloroquine was examined as described in A. All images are representative of three independent experiments. Scale bars: 1 mm (A, top panel in B,D); 200 μm (bottom panel in B,C).

To identify second-site mutations that suppress the *tpp1*^−^ developmental phenotype in the presence of chloroquine, we mutagenized cells using restriction enzyme-mediated integration (REMI) mutagenesis (Kuspa, 2006) and examined the development of mutant clones in the presence of chloroquine. Because REMI mutagenesis requires a parental strain that is sensitive to blasticidin, we generated a new strain, *tpp1flox*^−^, in which a blasticidin cassette had been inserted into the *ddTpp1* gene and then removed from the genome by using Cre-mediated recombination (Faix et al., 2004), generating a blasticidin-sensitive strain in which multiple in-frame stop codons are present within the ddTpp1 N-terminal domain (supplementary material Fig. S3). Like *tpp1*^−^ cells, *tpp1flox*^−^ cells showed aberrant development in the presence of chloroquine (Fig. 8A). We generated 30,000 REMI mutants in the *tpp1flox*^−^ background and examined the development of individual clones growing as colonies in the presence of chloroquine. We observed that one clone, which we named *stpA* (for suppressor of *tpp1*^−^ A), was able to form fruiting bodies under conditions where chloroquine was present, whereas the parental *tpp1flox*^−^ strain did not show fruiting body development (Fig. 8A).

We used inverse PCR analyses (Keim et al., 2004) to identify the genetic lesion in the *stpA* REMI strain, and we determined that the insertion was located within the coding region of the gene DDB_G0282973 (we subsequently refer to this gene as *stpA*). Although not a clear ortholog, StpA shows significant similarity to members of the OSBP family (supplementary material Fig. S4), which function in both the transcriptional regulation of lipid biosynthetic genes and the trafficking of lipids, such as sterols and sphingolipids (Olkkonen and Li, 2013). To test whether the suppression of the *tpp1*^−^ phenotype is due specifically to the loss of *stpA* function, we constructed a double mutant strain, *tpp1flox*^−^*/stpA*^−^, in which the entire *stpA* open reading frame had been deleted by using homologous recombination. We confirmed the knockout of the entire *stpA* gene in the *tpp1flox^−^* genetic background by using PCR analyses (supplementary material Fig. S5). *tpp1flox*^−^*/stpA*^−^ cells formed morphologically normal fruiting bodies under standard conditions (Fig. 8B, compare with Fig. 3B). Strikingly, and in contrast to *tpp1flox*^−^ cells, *tpp1flox*^−^*/stpA*^−^ cells did not show a delay in aggregation as compared with the wild type when starved in the presence of ammonium chloride (Fig. 8C). Further, whereas *tpp1flox*^−^ cells showed delayed development in the presence of chloroquine, *tpp1flox*^−^*/stpA*^−^ cells showed development similar to that of wild-type cells under these conditions (Fig. 8D). These results strongly suggest that loss of *stpA* function can suppress the hypersensitivity of *tpp1*^−^ cells to chloroquine and ammonium chloride during development. We then tested whether loss of *stpA* suppressed other phenotypes of *tpp1*^−^ cells. In *tpp1*^−^ cells, loss of *stpA* did not suppress the reduced cell size and viability of *tpp1*^−^ cells under autophagic conditions, the precocious development of *tpp1*^−^ at 12 hours, or the increased autofluorescence seen in *tpp1*^−^ cells (data not shown). These results indicate that loss of *stpA* function can suppress some but not all of the phenotypes of *tpp1*^−^ cells.

## DISCUSSION

Although mutations in the human protein TPP1/CLN2 underlie one of the NCL disorders, little is known about the physiological functions of this protein or how the loss of TPP1 function causes the symptoms associated with the disease. Here, we have shown that ddTpp1, an ortholog of TPP1, has multiple functional similarities to its human counterpart. Further, we identify a second-site suppressor mutation that suppresses some but not all of the phenotypes of *tpp1*^−^ cells, suggesting that the perturbation of second-site gene products could provide a therapeutic approach for NCL. Our work establishes *Dictyostelium* as a tractable system for the study of TPP1, which is absent in many traditional model organisms, such as *S. cerevisiae*, *Drosophila*, and *C. elegans*.

Although previous studies of TPP1 loss of function in other organisms have shown complete ablation of TPP1 peptidase activity, as measured by the degradation of a substrate *in vitro* (Awano et al., 2006; Mahmood et al., 2013; Passini et al., 2006; Walus et al., 2010), we saw that *tpp1*^−^ cells retained a significant amount of activity in this *in vitro* assay. Although we cannot rule out that our mutant is a hypomorph, we suspect that this residual TPP1 activity is due to a separate peptidase. In fact, a protein with TPP1 activity has been previously purified from *Dictyostelium* cell lysates (Krimper and Jones, 1999), although this purified protein showed a monomeric mass of 107 kDa, whereas mature ddTpp1 has a predicted mass of 42 kDa, suggesting that this purified protein was not ddTpp1. Further, the purified protein activity is most abundant at the vegetative life-cycle stage (Krimper and Jones, 1999), whereas ddTpp1 shows no detectable expression during the vegetative stage (Iranfar et al., 2001). The optimal pH for the activity of the purified peptidase is 4.5 (Krimper and Jones, 1999), suggesting that the peptidase is lysosomal. An exciting possibility is that an ortholog of this unidentified peptidase is present in humans but is not widely expressed and that therapeutic approaches that could upregulate this unidentified protease might serve to restore TPP1 activity. Thus, the identity of this peptidase is of considerable interest.

Consistent with the previously reported low expression of ddTpp1 in vegetative cells but high expression during development (Iranfar et al., 2001), we find that vegetative *tpp1*^−^ cells show no obvious phenotype, whereas starving or developing *tpp1*^−^ cells show substantial differences to wild-type cells. There are two possible explanations for why a lysosomal peptidase might be upregulated during starvation or development. First, starving or developing cells show little or no proliferation and generate cellular energy by using autophagy (Calvo-Garrido et al., 2010), and are thus in a position where they are generating potentially toxic catabolic byproducts while not being able to dilute these byproducts through continued proliferation. Thus, the expression of additional catabolic enzymes, such as ddTpp1, during development or starvation could be required to prevent the buildup of products of catabolism in non-proliferating cells, and this might explain why starved *tpp1*^−^ cells produce abnormally few spores. Interestingly, an analogous phenomenon occurs in cultured mouse cerebellum cells lacking the NCL-linked protein CLN3, where non-proliferating, confluent immortalized granule neurons that lack functional CLN3 show subcellular deposits of mitochondrial ATP synthase subunit c and autofluorescent material, whereas proliferating, sub-confluent cells of the same type do not show these hallmarks of NCL (Fossale et al., 2004). Furthermore, peptidases that are expressed during development might function in developmental signaling by acting on developmental signaling molecules or extracellular structural proteins (Fong and Bonner, 1979). We saw that *tpp1*^−^ cells showed precocious development, suggesting that ddTpp1 might in fact function in the negative regulation of development. Intriguingly, *Dictyostelium* cells lacking the ortholog of CLN3, another gene that can cause NCL when mutated, also showed precocious development at the mound stage (Huber et al., 2014). It could be the case that in *Dictyostelium,* NCL-associated genes play a significant role in developmental timing. Investigation of other NCL genes [the *Dictyostelium* genome encodes orthologs of CLN1-5, CLN7 and CLN10-14 (Huber et al., 2014)] will reveal the extent of this functional overlap. One possibility is that NCL-linked genes might affect the activity of the protein kinase A (PKA) pathway, which is crucial for proper developmental timing (Loomis, 1998).

We found that the loss of ddTpp1 affected cell size and survival under autophagy-stimulating conditions, suggesting that ddTpp1 affects the autophagic response. Cells that are unable to undergo autophagy do not show a significant reduction in size during autophagic conditions, but they do show reduced viability during autophagic conditions, suggesting that aberrant autophagy can cause a loss in viability due to an inability to generate nutrients through catabolism (King et al., 2013). *tpp1*^−^ cells do show a reduction in size in autophagy-stimulating conditions, indicating that autophagy is not abolished but is aberrant in these cells. The abnormally rapid reduction in cell size and cell viability of *tpp1*^−^ cells under autophagy-stimulating conditions indicates the possibility that the *tpp1*^−^ cells have enhanced autophagy, and thus that one function of ddTpp1 might be to limit autophagy. Autophagy has previously been shown to be aberrant in NCL – mice mutant for CLN3 show an increase in the autophagic marker LC3 in brain tissue (Cao et al., 2006), and CLN2 mutant human fibroblasts under autophagic conditions show significantly higher levels of reactive oxygen species than wild-type cells (Vidal-Donet et al., 2013). Future studies in *Dictyostelium* might help to reveal the role of autophagy in the reduced viability of cells that lack TPP1.

The development of *tpp1*^−^ cells is strongly inhibited by chloroquine, and this phenotype is suppressed by a secondary mutation in StpA, a protein with similarity to mammalian OSBPs. OSBPs function in the subcellular trafficking of lipids, and the regulation of sterol and sphingolipid metabolism (Olkkonen and Li, 2013). Intriguingly, many NCL genes have been linked to sphingolipid metabolism (Granier et al., 2000; Haltia et al., 1995; Persaud-Sawin et al., 2004; Persaud-Sawin et al., 2007), and cerebral samples from individuals with mutations in the *CLN8* gene show reduced levels of the sphingolipid ceramide (Hermansson et al., 2005). An exciting possibility is that the modulation of sphingolipid metabolism by targeting sphingolipid regulators, such as OSBPs, might be a way of controlling the pathogenic effects of NCL.

*Dictyostelium* has proven to be a useful system for studying proteins that are associated with lysosomal (Maniak, 2011) and neurodegenerative diseases (Annesley et al., 2014; Myre et al., 2011). We have demonstrated that the *Dictyostelium* ortholog of TPP1 shows multiple functional parallels with the human protein and that the genetic tools available in *Dictyostelium* can be used to find genetic suppressors of phenotypes that are caused by loss of ddTpp1 function. Future studies in *Dictyostelium* might reveal additional genes that are functionally linked with ddTpp1 and will provide a better understanding of the physiological functions of TPP1, which is crucial for developing future therapies for NCL.

## MATERIALS AND METHODS

### Cell culture and strain construction

Cells were maintained in axenic shaking culture in HL5 medium (Formedium Ltd, Norwich, UK) at 20°C. All strains described in this study are derived from the Ax-2 strain. *Dictyostelium* transformations were performed following standard protocols (Gaudet et al., 2007). Examination of development on agar plates was performed as described previously (Tang and Gomer, 2008). Proliferation curves were generated as described previously (Brock and Gomer, 2005), except that cells were inoculated at 1×10^5^ cells/ml. Spore viability assays were performed as described previously (Phillips and Gomer, 2010). For each independent experiment, spores for each genotype were collected from fruiting bodies of the same age.

Previously, the *ddTpp1* gene had been disrupted by using REMI mutagenesis at genomic position Chr1:3853302, resulting in the insertion of a blasticidin-resistance cassette and multiple in-frame stop codons within the sequence encoding the N-terminal pro-peptidase activation domain of ddTpp1. A plasmid containing this cassette and flanking genomic arms from the insertion site had been previously cloned into bacteria by using plasmid rescue (the isolated plasmid was a gift from Chris Dinh and Adam Kuspa, Baylor College of Medicine, Houston, Texas), allowing recreation of this mutation by using homologous recombination. To generate a *ddTpp1* disruption mutant, the *ddTpp1* REMI vector was linearized using *Eco*RI digestion and was then transformed into wild-type cells. To confirm disruption of the *ddTpp1* locus, we used PCR to show the absence of an intact locus using the genomic primers 5′-TCACGATCCAAGTGGGTACA-3′ and 5′-GGCGATTTCATTCGTACCAT-3′, and the presence of the REMI insertion at the *ddTpp1* locus using the primers 5′-GAGTTGGAAGATTTCGTGTG-3′ and 5′-ATTTAGGTGACACTATAG-3′ (supplementary material Fig. S2).

To generate a *ddTpp1* rescue strain expressing Tpp1 under control of the *cotB* promoter, we used the plasmid pVSC (Zhang et al., 1999), a gift from Chris West (University of Oklahoma College of Medicine, Oklahoma City, OK, USA). The *ddTpp1* open reading frame, excluding the stop codon, was amplified from genomic *Dictyostelium* DNA (*ddTpp1* has no introns) using the primers 5′-GGGTACCATGAATATTAAATTTAATTTAATAATTATAA-3′ and 5′-GAGATCTTTCAAGACAATATTTAACCAATTC-3′, which generated *Kpn*I and *Bgl*II sites at the start and terminus of the open reading frame, respectively. This PCR fragment was then digested with *Kpn*I and *Bgl*II and cloned into pVSC, and the resulting plasmid, pJP105, was transformed into *tpp1*^−^ cells. To express human TPP1 in *Dictyostelium*, the human TPP1 open reading frame lacking the terminal stop codon was amplified by using PCR of cloned human TPP1 cDNA (a kind gift from Peter Lobel, Rutgers University, Highland Park, NJ, USA) using the primers 5′-GGGTACCATGGGACTCCAAGCCTGC-3′ and 5′-GAGATCTGGGGTTGAGTAGAGTCTTCAGC-3′, which generate *Kpn*I and *Bgl*II sites at the start and terminus of the open reading frame, respectively. This PCR product was cloned into pVSC and the resulting plasmid pJP106 was transformed into *tpp1*^−^ cells. To generate cells expressing ddTpp1-GFP, the *ddTpp1* open reading frame lacking the stop codon was amplified from genomic DNA by PCR with the primers 5′-GGAGATCTATGGGACTCCAAGCCTGC-3′ and 5′-GGACTAGTGGGGTTGAGTAGAGTCTTCAGC-3′, which add *Bgl*II and *Spe*I sites to the ddTPP1 start and terminus, respectively. The PCR product was digested with *Bgl*II and *Spe*I and then ligated into the vector pDM323 (Veltman et al., 2009), generating an open reading frame encoding ddTpp1 with a C-terminal GFP fusion. The resulting plasmid pJP107 was then transformed into *tpp1*^−^ cells. To generate *Dictyostelium* cells expressing human TPP1-GFP, an identical strategy was used, except that human TPP1 was amplified from cloned cDNA using the primers 5′-GGAGATCTATGGGACTCCAAGCCTGC-3′ and 5′-GGACTAGTGGGGTTGAGTAGAGTCTTCAGC-3′.

To generate a ddTpp1 disruption vector in which LoxP sites flank the blasticidin resistance cassette, we cloned the homologous arms from the ddTpp1 REMI vector into the vector pLPBLP (Faix et al., 2004). The primers 5′-GGGGTACCACCATTATTGTCACCCAGACC-3′ and 5′-GGAAGCTTATAAAGTTCTCTTGGAATAGTGTAAGG-3′ were used to amplify the homologous arms and the connecting *Eco*RI site, generating a DNA fragment with the following components: *Kpn*I site – Homologous arm 1 – *Eco*RI site – Homologous arm 2 – *Hin*dIII site. This fragment was digested with *Kpn*I and *Hin*dIII and cloned into the *Kpn*I and *Hin*dIII sites of pLPBLP. The generated vector pJP108 was then digested with *Eco*RI, yielding a single DNA fragment flanked by arms homologous to the *tpp1* locus, and transformed into Ax2 cells. To confirm disruption of the *ddTpp1* gene, we tested the absence of an intact *ddTpp1* gene with the genomic PCR primers 5′-GAGTTGGAAGATTTCGTGTG-3′ and 5′-TCACGATCCAAGTGGGTACA-3′, and the presence of the insert at the *ddTpp1* locus with the PCR primers 5′-GAGTTGGAAGATTTCGTGTG-3′ and 5′-AGCATTGTAATCTTCTCTGTCG-3′. To generate the blasticidin-sensitive strain *tpp1flox*^−^, the blasticidin-resistance cassette was removed by using Cre-mediated recombination (Linkner et al., 2012). To confirm blasticidin cassette removal, we tested blasticidin-sensitive clones using the genomic PCR primers 5′-GGCGATTTCATTCGTACCAT-3′ and 5′-TCACGATCCAAGTGGGTACA-3′, and confirmed that the product was the size expected for successful excision of the blasticidin S resistance gene (BSR) by using recombination (supplementary material Fig. S3).

To generate a *tpp1flox*^−^*/stpA*^−^ strain, homologous arms flanking the entire *StpA* (DDB_G0282973) open reading frame were amplified by using PCR from genomic DNA using the primer pairs 5′-GGGGTACCATCACCACAACCATCAAATGC-3′, 5′-GGAAGCTTAATAAATGCAAATTAATTACTGATGG-3′ and 5′-CCGGATCCTTATATTGTATAATTGGTTCACTTTACC-3′, 5′-GGACTAGTCCCCCTATAAGTTCTAAATAAACAG-3′. These primers incorporate terminal restriction sites for *Kpn*I, *Hin*dIII, *Bam*HI and *Spe*I, respectively. These PCR products were cloned into the vector pLPBLP, generating the plasmid pStpA-KO. pStpA-KO was digested with *Kpn*I and *Spe*I and transformed into *tpp1flox*^−^ cells. To confirm disruption of the *StpA* gene, we used the genomic primers 5′-TGCTGCTGGTACTAGAACTTCG-3′ and 5′-TTTTTCTGCATATTTGTGTGG-3′ to show the lack of an intact *StpA* gene, and the primers 5′-TGCTGCTGGTACTAGAACTTCG-3′ and 5′-CACTCGAATACTTCTATCTACTTCGTC-3′ to show that the blasticidin-resistance cassette had replaced the *StpA* open reading fame (supplementary material Fig. S5).

### Tpp1 enzymatic assays

To measure Tpp1 activity in cell lysates, 3 ml of cells at 5×10^6^ cells/ml were starved for 16 hours in PBM buffer (20 mM KH_2_PO_4_, 0.01 mM CaCl_2_, 1 mM MgCl_2_, pH 6.1 with KOH). Cells were then counted, resuspended at 7.5×10^6^ cells/ml in 0.05 M MES pH 6.5 with 0.1% NP-40, and lysed by passage through a Cameo 5 μm nylon filter (Spectrum, New Brunswick, NJ, USA) using a 1 ml syringe. A solution of 100 μM TPP1 substrate AAF-AMC HCL (Bachem) was made in 100 mM sodium acetate pH 3.8, and 50 μl of cell lysate (or, as a control, 50 μl of buffer) was mixed with 50 μl of AAP-AMC solution in a 96-well opaque plate (Nunc) to give a final concentration of 50 μM Ala-Ala-Phe-AMC. Plates were then incubated at room temperature for 30 minutes in the dark, and the fluorescence of the released amino methyl coumarin (AMC) was measured using a Synergy MX microplate reader (Biotek) using excitation at 360 nm and emission at 440 nm. The fluorescence value for the buffer control condition was subtracted from the fluorescence values for the experimental samples, and then values were normalized to the wild-type condition for each independent experiment.

### Microscopy and fluorescence

To examine Tpp1-GFP localization, cells at 2×10^6^ cells/ml in PBM were starved in shaking culture for 14 hours, and then cells were collected by centrifugation and resuspended in PBM containing 500 nM Lysotracker Red (Invitrogen), and incubated in shaking culture for 2 hours. Cells (50 μl) were then allowed to settle in a well of an 8-well glass chamber slide (Nunc) for 20 minutes, and the medium was removed and replaced with 200 μl PBM. Cells were then imaged by using an Olympus FV1000 confocal microscope. To examine the autofluorescence of cells by microscopy, cells growing in shaking culture in HL5 medium were resuspended in PBM at 1×10^6^ cells/ml, and then 200 μl of the cell suspension was allowed to settle in one well of an 8-well glass-bottomed chamber slide (Nunc). Cells were incubated at 20°C for 16 hours, and then the autofluorescence of live cells at the periphery of cell aggregates was imaged by using an Olympus FV1000 confocal microscope with excitation with a 543-nm laser and detection of fluorescence at 555–655 nm. To measure cellular autofluorescence by using flow cytometry, cells growing in shaking culture in HL5 medium were resuspended in PBM at 1×10^6^ cells/ml, and were incubated for 16 hours at 20°C on a rotary shaker. Cells were then vortexed and analyzed by using flow cytometry with a C6 flow cytometer (Accuri) using a 488-nm excitation laser and a 670-nm LP (FL-3) filter to detect autofluorescence. For each independent experiment, the mean autofluorescence of the population was calculated and normalized to the wild-type value. To examine the autofluorescence of vegetative cells by using flow cytometry, 1×10^3^ cells were mixed with *K. aerogenes* bacteria and plated onto SM/5 plates. After 48 hours at 20°C, *Dictyostelium* cells and bacteria were washed from the plate and suspended in 10 ml PBM, and cells were washed twice by pelleting at 500 ***g*** and resuspending in 10 ml PBM to remove most of the bacteria. An aliquot of the cell suspension was then analyzed for autofluorescence by flow cytometry as described above, with any remaining bacteria excluded from the analysis by gating based on forward scatter.

### Cell size and viability in media lacking arginine and lysine

Log-phase cells growing in HL5 medium in shaking culture were washed twice in SIH medium lacking arginine and lysine (ASM medium) (Formedium Ltd, Norwich, UK) and then resuspended to 5×10^6^ cells/ml in 5 ml ASM. An aliquot of cells was taken from the culture daily for flow cytometry analysis using an Accuri C6 flow cytometer, and the average forward scatter of the cell population was measured. To convert forward scatter measurements into microns, we measured the forward scatter of beads with diameters of 3.27, 5.05 and 10.1 microns (Spherotech), generated a best-fit curve, and used the corresponding equation to convert the forward scatter values to diameter in microns. To assay viability, an aliquot of cells from a culture started at 5×10^6^ cells/ml in 5 ml of ASM was taken every 2 days, and serial dilutions were plated with *K. aerogenes* bacteria onto SM/5 plates. Counts of the resultant colonies were used to calculate the percent cell survival of the culture by dividing the observed colony count by the theoretical predicted colony count if cell density had not changed since inoculation and if every cell from the original inoculation had generated a colony.

### Development and aggregation in the presence of chloroquine or ammonium chloride

To examine development in the presence of chloroquine, SM/5 agar was prepared, and chloroquine (Sigma-Aldrich) was added to a final concentration of 3 mM 30 minutes after autoclaving, while the agar was still molten. SM/5 containing 3 mM chloroquine was then poured into Petri plates in 24-ml volumes. Serial dilutions of cells were spread with *K. aerogenes* onto the chloroquine-containing plates. After 8 days, well-spaced colonies were imaged either from overhead using a camera stand with side illumination or with an inverted microscope equipped with a 2× objective, allowing for imaging through the agar. To examine aggregation in submerged culture, log-phase cells growing in HL5 medium in shaking culture were washed in PBM and then resuspended at 1×10^6^ cells/ml in PBM, PBM with 5 mM chloroquine, or PBM with 20 mM ammonium chloride. Cells were then pipetted into either 24-well plates in 1-ml volumes or 384-well plates in 57-μl volumes. After a 14-hour room temperature incubation, cells were imaged using an inverted microscope.

### REMI mutagenesis

REMI mutagenesis was performed using the plasmid pBSR1 as described previously (Kuspa, 2006). Inverse PCR was performed as described previously (Keim et al., 2004). To screen for suppressor mutations of the *tpp1flox*^−^ phenotype, *tpp1flox*^−^ cells were mutagenized, and pools of REMI mutants were plated onto plates containing SM/5 with 3 mM chloroquine, as described above. Wild-type and *tpp1flox*^−^ cells were plated in parallel as controls. REMI mutant clones that showed more rapid fruiting body development as compared with the parental strain were selected for further analysis.

### Statistics and alignments

Statistical analyses were performed using Prism (GraphPad Software). Significance was defined as *P*<0.05. To generate alignments, sequences were aligned using the CLUSTALW algorithm (Thompson et al., 1994), and the alignments were formatted using the program BOXSHADE (written by K. Hoffman and M. Baron).

## Supplementary Material

Supplementary Material
