# Supplementary material for: Partial genetic suppression of a loss-of-function mutant of the neuronal ceroid lipofuscinosis-associated protease TPP1 in Dictyostelium discoideum
Source: Dis Model Mech. 2014 Dec 24;8(2):147–56. doi: 10.1242/dmm.018820 (PMC4314780; doi:10.1242/dmm.018820)
Supplement: Supplementary Material [file supp_8_2_147__index.html]

Partial genetic suppression of a loss-of-function mutant of the neuronal ceroid lipofuscinosis-associated protease TPP1 in Dictyostelium discoideum — Supplementary Material 

# Partial genetic suppression of a loss-of-function mutant of the neuronal ceroid lipofuscinosis-associated protease TPP1 in *Dictyostelium discoideum*

## DMM018820 Supplementary Material

**Files in this Data Supplement:**

- **Supplementary Material**
