# Supplementary material for: Partial genetic suppression of a loss-of-function mutant of the neuronal ceroid lipofuscinosis-associated protease TPP1 in Dictyostelium discoideum
Source: Dis Model Mech. 2014 Dec 24;8(2):147–56. doi: 10.1242/dmm.018820 (PMC4314780; doi:10.1242/dmm.018820)
Supplement: Supplementary Material [file supp_8.2.147_DMM018820.pdf]

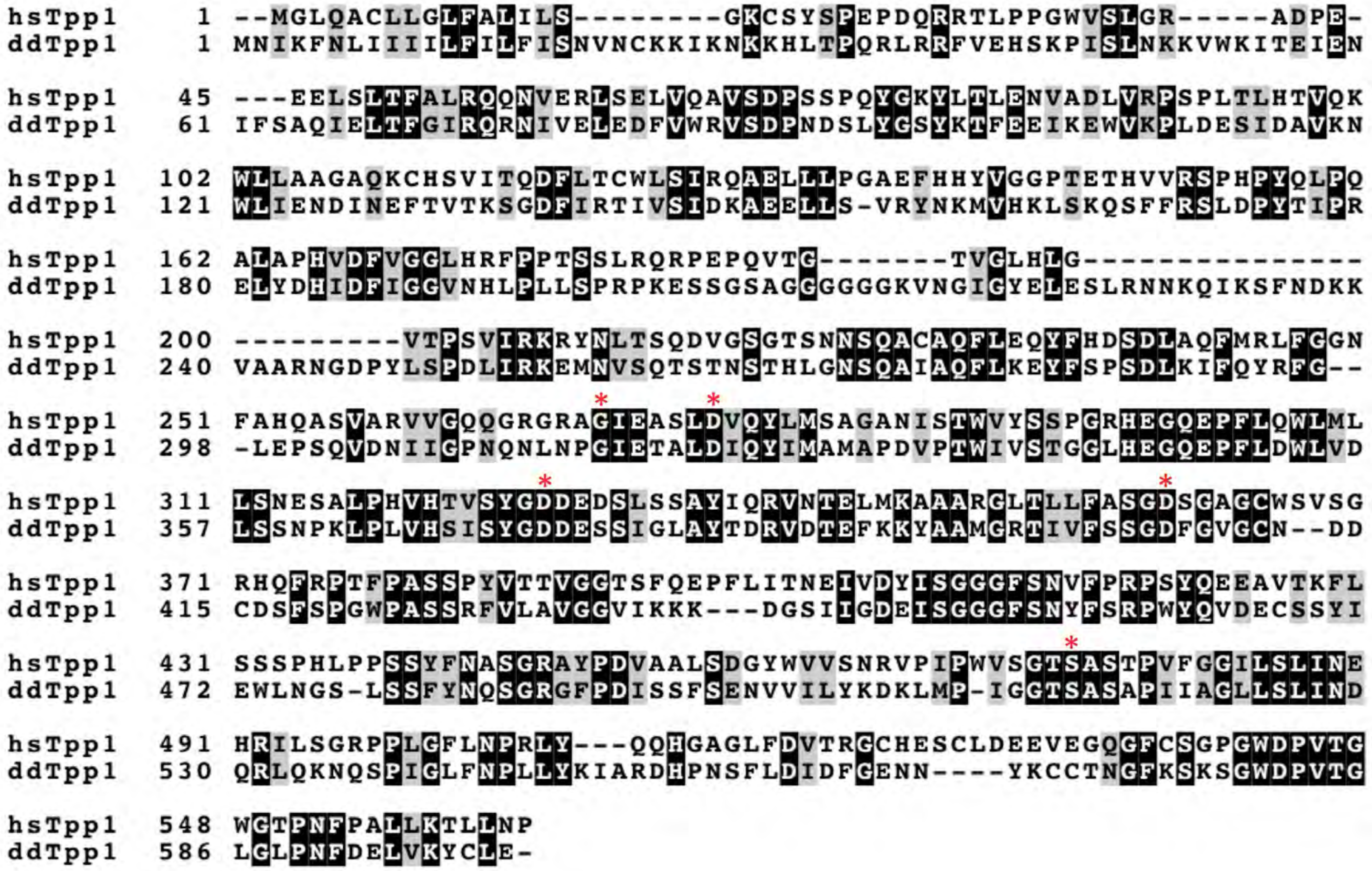

Fig. S1. Human and *Dictyostelium* Tpp1 are similar and show conservation of catalytic residues. An alignment of the complete sequences of human (hs) and *Dictyostelium* (dd) Tpp1 is shown. Identical residues are highlighted in black, and similar residues are in grey. Residues required for catalytic activity of human Tpp1 (see main text) are marked with a red asterisk.

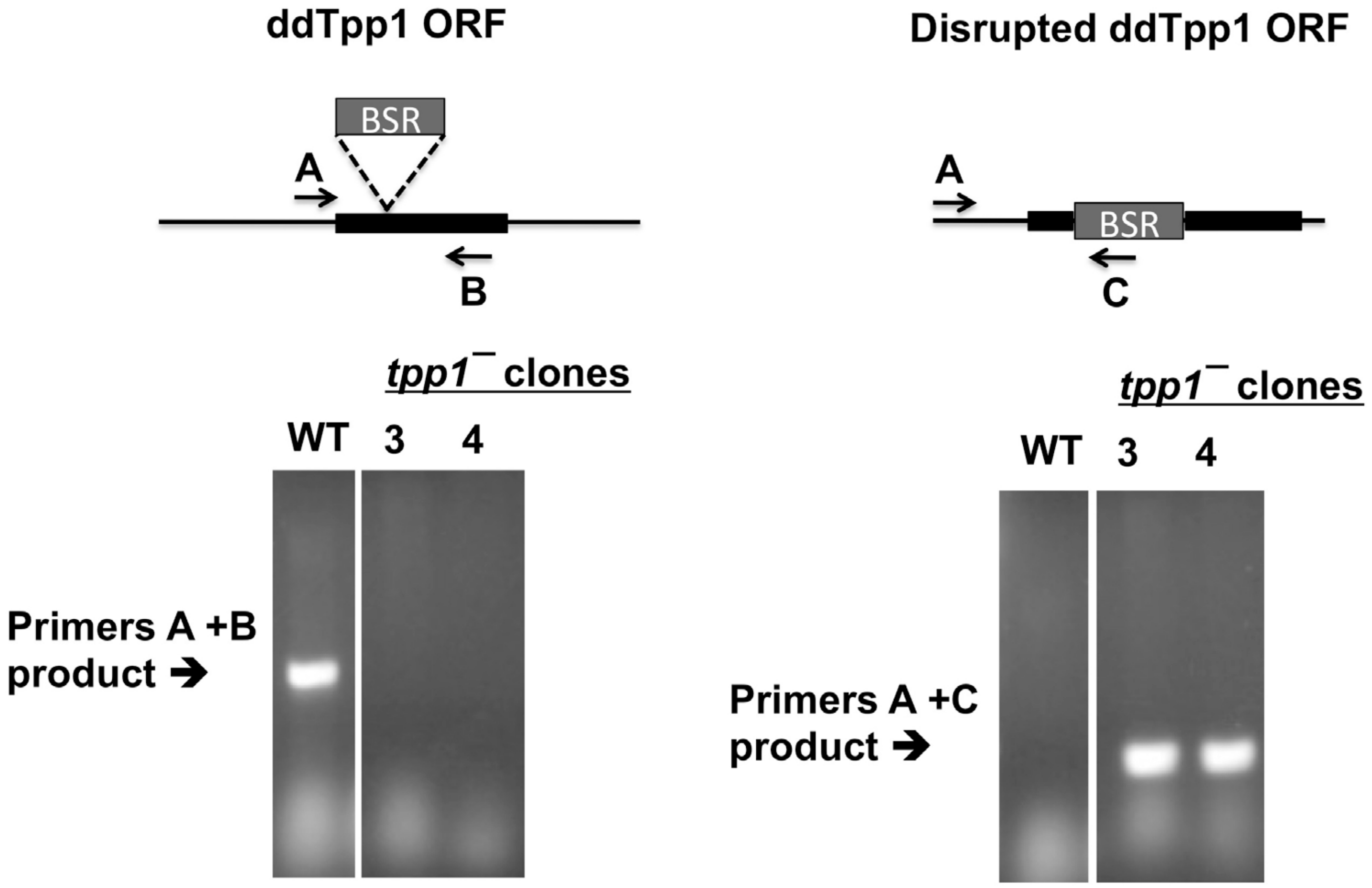

Fig. S2. The ddTpp1 gene is disrupted in *tpp1*‾ clones. Wild-type Ax2 cells were transformed with the ddTpp1 disruption plasmid (described in text) and individual clones were tested for disruption of the ddTpp1 gene by PCR as described in Materials and Methods (a short extension time prevented amplification across the BSR insertion). Genomic DNA preparation, PCR reactions, and gel electrophoresis were done in parallel for all cell lines. “BSR” indicates blasticidin resistance cassette.

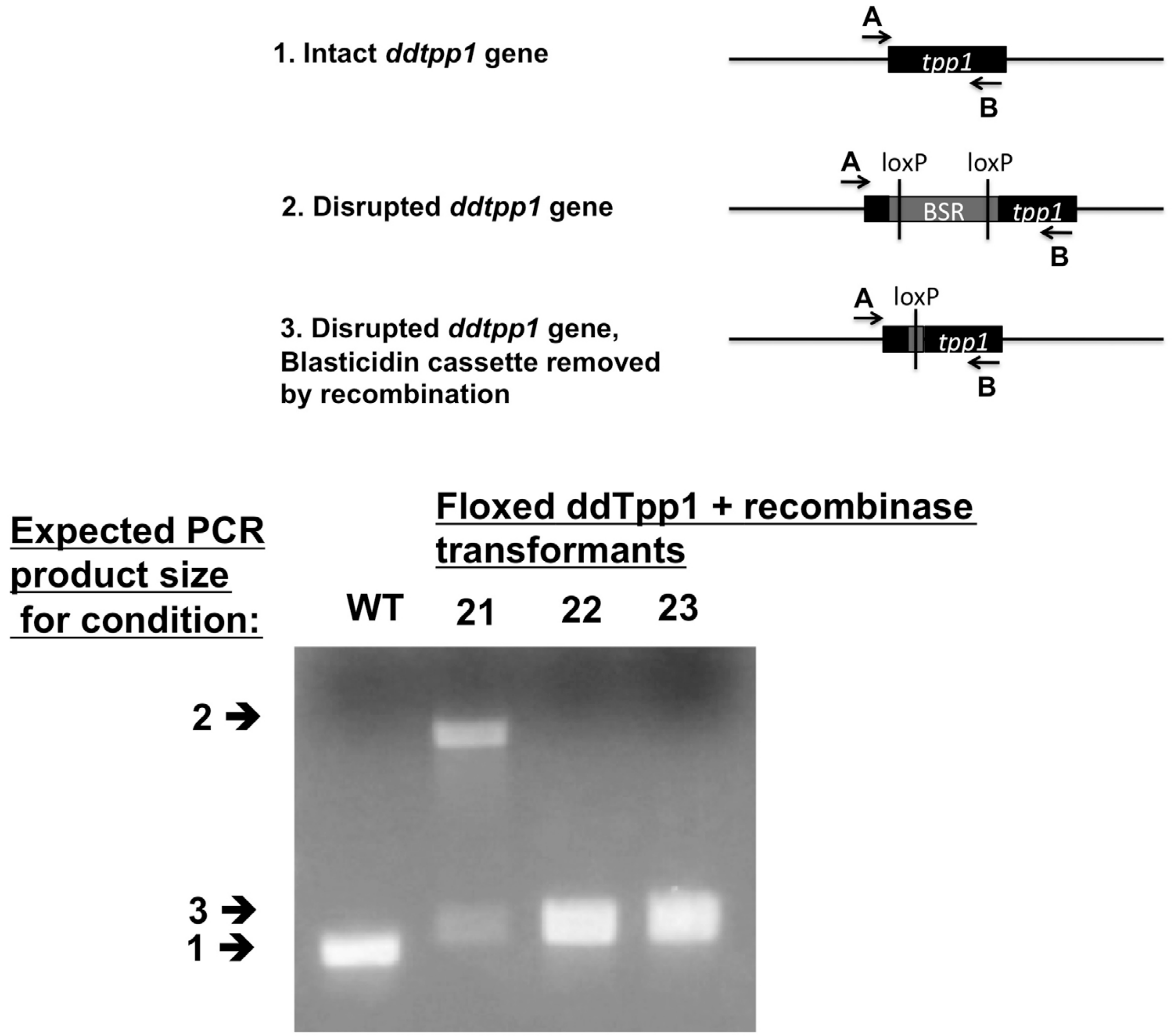

Fig. S3. Construction of the Blasticidin-sensitive *tppflox*$^{-}$ strain. *ddtpp1* was disrupted with a loxP-flanked blasticidin resistance cassette (BSR) by homologous recombination. This strain was then transformed with pTX-NLS-CRE (Linkner et al., 2012), and transformants that were sensitive to Blasticidin were tested for loss of the BSR cassette by PCR. Clones 22 and 23 show a PCR product size indicating successful recombination. The two visible PCR products in clone 21 indicate a mixed population where recombination has occurred in a subset of cells. Clone 22 was designated "*tpp1flox*$^{-}$" and was used for subsequent experiments. Genomic DNA preparation, PCR reactions, and gel electrophoresis were done in parallel for all cell lines.

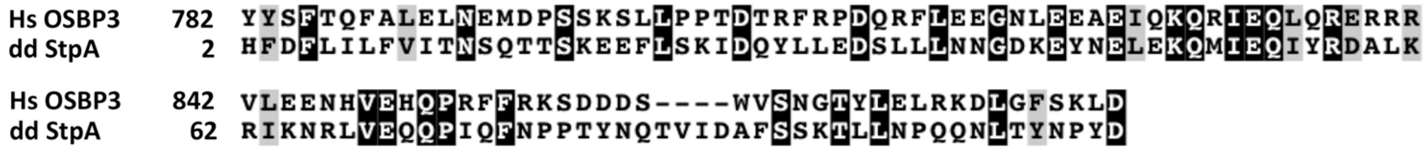

Fig. S4. StpA shows similarity to human oxysterol-binding proteins. A bioinformatic search using the BLAST algorithm indicated that StpA shows significant similarity to the human oxysterol-binding protein 3 (OSBP3) (E value = 0.019). The segment of OSBP3 shown corresponds to a sequence near the C-terminal end of the protein located within the conserved OSBP-related ligand-binding domain (ORD) found within OSBP3. An alignment between selected regions of each protein is shown. Identical residues are highlighted in black, and similar residues are in grey.

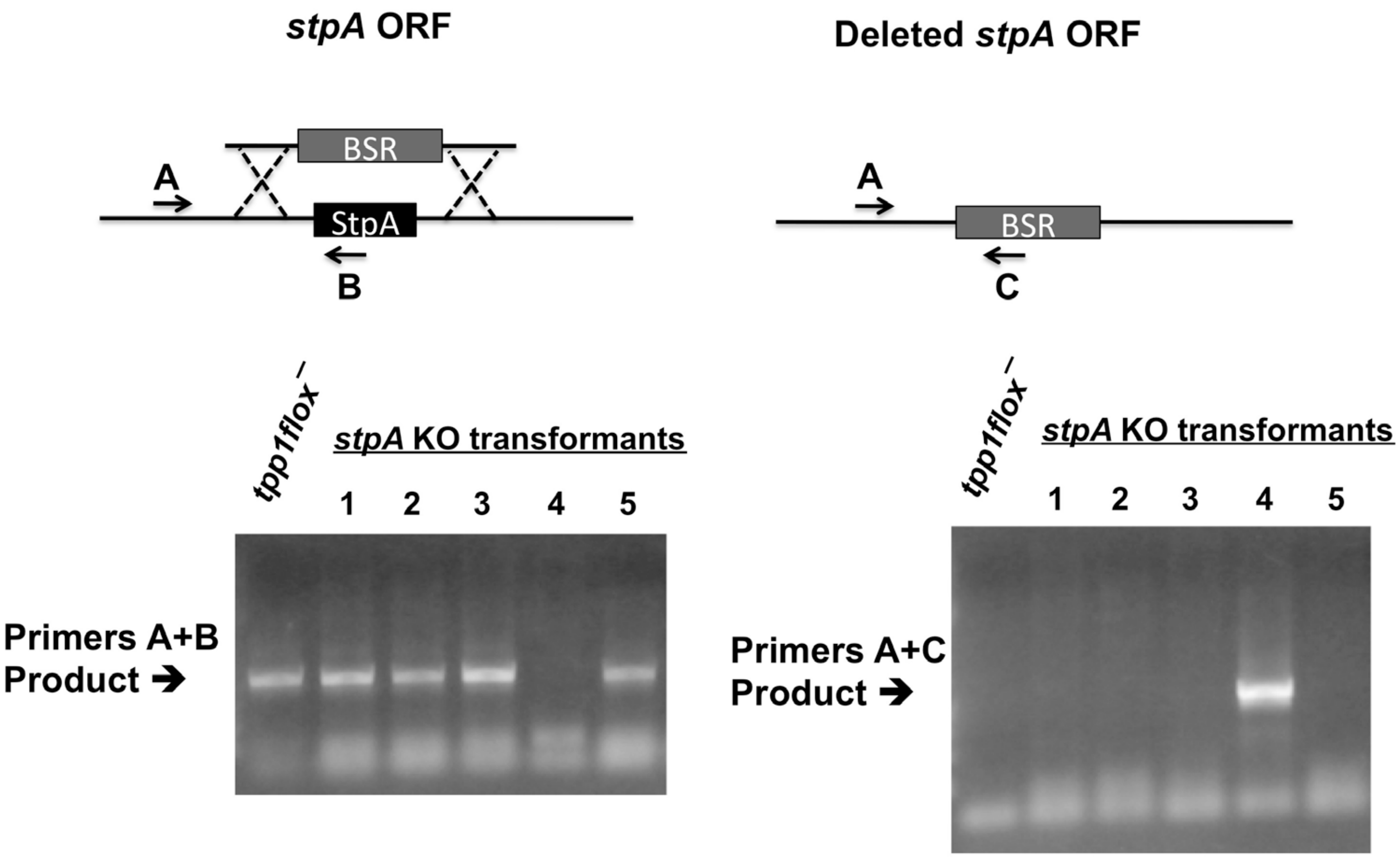

Fig. S5. Generation of the *tpp1flox*‾/*stpA*‾ double mutant. *tpp1flox*‾ cells were transformed with the *stpA* knockout construct as described in the Materials and Methods. Transformant clones were examined for successful knockout of the *stpA* gene with the indicated primers A, B, and C. Transformant 4 shows the predicted PCR products for successful knockout of *stpA*, and was designated *tpp1flox*‾/*stpA*‾ and used in subsequent experiments. Dashed lines indicate homologous recombination between matching sequences.
